# Editorial: Novel approaches to rapid diagnosis and treatment monitoring of active tuberculosis, vol II

**DOI:** 10.3389/fmicb.2022.1044314

**Published:** 2022-10-07

**Authors:** Zhidong Hu, Xiao-Yong Fan

**Affiliations:** Shanghai Public Health Clinical Center, Fudan University, Shanghai, China

**Keywords:** *Mycobacterium tuberculosis*, molecular diagnoses, biomarker, tuberculosis, latent infection

Tuberculosis (TB), caused by respiratory *Mycobacterium tuberculosis* (*Mtb*) infection, remains a major global threat to human health. According to the World Health Organization (WHO)'s latest Global Tuberculosis Report, there were an estimated more than 10 million new TB cases and 1.4 million deaths in 2020 (WHO, 2021). Considering TB transmission have already happened before the diagnosis progress of passive case findings, the rapid and reliable test to detect active *Mtb* infections are needed, especially the diagnosis of the emerging drug-resistant strains (Dheda et al., 2019; Gunther et al., 2022). Detection of active TB patients, monitoring of therapy and determination of treatment effect are significant challenge for TB control efforts, the currently routine TB diagnostic tools, including *Mtb* culture, acid-fast staining, tuberculin skin test, chest X-ray, GeneXpert MTB/RIF, etc., all have their limitations (Al-Zamel, 2009; Heyckendorf et al., 2022). This challenge is complicated by the fact that sputum sample collection is not always straightforward to obtain, especially in TB patients with improved symptom, extrapulmonary TB (EPTB) patients, and childhood TB (Norbis et al., 2014; Jonckheree and Furin, 2017). The GeneXpert MTB/RIF sputum assay based on PCR was developed to improve the specificity and speed of diagnosis, but it cannot distinguish between viable and non-viable bacilli, and its sensitivity under low bacterial loads is still waiting to be optimized. The challenges of GeneXpert MTB/RIF assay to monitoring the treatment effect is also existed, especially in patients co-infected with HIV and TB (Denkinger et al., 2014; Detjen et al., 2015; Naidoo and Dookie, 2022).

In this Research Topic, we summarize a number of studies that either investigated novel methods in the diagnosis of TB or optimized the current TB diagnosis assays, and several reviews that focused on different topics and indicated directions for future research.

Jiao et al. determined the diagnostic efficacy of multiple cross displacement amplification (MCDA), which is a DNA amplification strategy on the basis of an isothermal strand-displacement polymerization reaction, and combined it with real-time PCR assay in patients with pulmonary TB. The MCDA assay showed a higher sensitivity than microscopy, culture, or Xpert using sputum samples, with a slight drop in specificity. Thus, MCDA assay might assist in the accurate and rapid diagnosis of TB in settings with platforms of real-time PCR equipment. Quan et al. evaluated the diagnostic efficacy of EasyNAT MTB complex assay (EasyNAT), which is a novel cross-priming amplification-based by using gastric aspirate samples in childhood TB. Compared with Xpert Ultra, EasyNAT yielded similar specificity but a modest lower sensitivity in childhood TB diagnosis using gastric aspirate samples. Thus, EasyNAT might be used as an alternative method for diagnosing childhood TB due to its cost-effectiveness and speed. Wang et al. conducted a prospective, multicenter study of the plasma concentrations of soluble triggering receptors expressed on myeloid cells (sTREM)-1 and sTREM-2 in subjects undergoing 3HP treatment regimen (once-weekly rifapentine plus isoniazid for 3 months) and examined the use of these biomarkers to predict systemic adverse reactions (SARs). The baseline concentrations of sTREM-1 were higher in patients with SARs than those without, and the area under the receiver operating characteristic curves showed that the plasma levels of sTREM-1 and sTREM-2 had modest discriminative power pertaining to the development of SARs during 3HP treatment at day 1. Pan et al. evaluated the performance of a novel *Mtb*-specific *CXCL10* mRNA release assay in TB diagnosis. The *CXCL10* gene encodes the IP-10 protein, which was shown to have potential as a biomarker of *Mtb* infection due to its high expression level after *Mtb* antigen stimulation. Their assay provided a sensitivity of 93.9% and a specificity of 98.0% in the diagnosis of *Mtb* infection, respectively, similar to T-SPOT.TB which gave a sensitivity of 94.5% and a specificity of 100%. Díaz-Fernández et al. compared the capacity of cell surface markers CD27, CD38, HLA-DR, and Ki-67 to distinguish LTBI, active TB, and patients who ended treatment and resolved TB. Their study showed that the percentages of cells bearing CD27^−^, CD38^+^, HLA-DR^+^, and Ki-67^+^ on *Mtb*-specific CD4 T cells were increased during the progression of active TB disease, and unbiased multiparametric analyses identified cell clusters based on CD27 or HLA-DR whose abundance can be correlated to treatment efficacy. These novel methods showed potential clinical application.

The study of Antonello et al. determined the performances of in-house droplet digital PCR (ddPCR)-based assays compared to culture using 89 biopsies, including fresh and formalin-fixed and paraffin-embedded (FFPE) samples. Their analysis support a highly accurate, sensitive, and rapid *Mtb* diagnosis with FFPE samples by ddPCR assay, as defined by a high concordance between the IS6110 assay and culture results. The quantitative method of ddPCR assay could differentiate a high bacillary load and disseminated condition from paucibacillary anatomically compartmentalized TB; this might accelerate *Mtb* diagnosis when culture techniques are unavailable. Carrère-Kremer et al. analyzed the cytokines patterns in QuantiFERON Gold Plus assay-positive populations. Their data showed that higher IFN-γ responses, and lower ratios of tube 1/tube 2 IFN-γ concentrations, were measured in the active TB group compared with LTBI. Patients with low ratios of IL-2/IFN-γ, IP-10/IFN-γ, and MIG/IFN-γ were much more likely to have active TB. These features of T cell response may be helpful in low prevalence settings to raise suspicion of ATB in patients who tested positive in IFN-γ release assays. Thus, these studies might optimize the current TB diagnosis assays.

Lin et al. tested the performance of endobronchial ultrasound-guided transbronchial biopsy (EBUS-TBB) in TB diagnosis and showed it to be safe and effective in diagnosing sputum smear-negative pulmonary TB. The EBUS echoic feature was also a predictor of the positive TB culture rate. To improve the efficiency of clinical diagnosis in spinal TB patients by computed tomography (CT), Li et al. developed a novel deep learning method based on three handcrafted features and one convolutional neural network feature. This gave efficient feature fusion for multimodal features. The introduction of these methods into the TB research field provides novel approaches for TB diagnosis.

Rapid TB diagnosis is challenging in EPTB infection due to the small number of mycobacteria and the lack of fresh samples with which to apply culture techniques. Rindi reviewed the current knowledge of the diagnostic performance of the Xpert Ultra assay in EPTB detection. It was shown that the sensitivity of the Xpert Ultra assay differs by specimen types, with high sensitivity among specimens obtained from cerebrospinal and fluid lymph nodes, and low sensitivity when using pleural fluids. Similar challenges are faced in the diagnosis of latent TB and the review by Gong et al. focused on this. Firstly, the authors summarized the concept and expounded on the immunological mechanism of LTBI. Secondly, they outlined novel interferon-gamma release assays and skin tests that have been developed recently. Finally, they summarized the research status, directions, and challenges in LTBI diagnosis, including novel biomarkers, new models/algorithms, omics technologies, and microbiota. The review written by Guo et al. focused on the progress of proteomics in biomarker discovery in TB diagnosis. Firstly, the authors summarized the proteomics research approaches. Secondly, the current status of research on the diagnostic application of proteomic biomarkers for TB was outlined. Finally, they described the prospects of proteomics in the field of TB biomarker discovery for rapid and accurate TB diagnosis. These high-quality reviews gave a thorough and up-to-date summary of their topics and provided directions for future research.

Overall, the manuscripts reviewed within this Research Topic demonstrate that the field of TB diagnosis is now rapidly evolving, providing us with new insights into how we can increase the sensitivity and specificity of TB diagnosis. Novel research approaches are indicated that may further improve the diagnosis of active TB cases, the identification of latent TB, of susceptible and resistant isolates of *Mtb*, and enable better monitoring the efficacy of anti-TB treatment in near future.

## Author contributions

ZH and X-YF conceived, designed, and wrote the manuscript. X-YF edited the manuscript with conceptual advice. Both authors contributed to the article and approved the submitted version.

## Conflict of interest

The authors declare that the research was conducted in the absence of any commercial or financial relationships that could be construed as a potential conflict of interest.

## Publisher's note

All claims expressed in this article are solely those of the authors and do not necessarily represent those of their affiliated organizations, or those of the publisher, the editors and the reviewers. Any product that may be evaluated in this article, or claim that may be made by its manufacturer, is not guaranteed or endorsed by the publisher.
